# Network assortativity for a multidimensional evaluation of socio-economic territorial biases in university rankings

**DOI:** 10.1371/journal.pone.0323356

**Published:** 2025-06-10

**Authors:** Loredana Bellantuono, Andrea Lo Sasso, Nicola Amoroso, Alfonso Monaco, Sabina Tangaro, Roberto Bellotti

**Affiliations:** 1 Università degli Studi di Bari Aldo Moro, Dipartimento di Biomedicina Traslazionale e Neuroscienze (DiBraiN), Bari, Italy; 2 Istituto Nazionale di Fisica Nucleare, Sezione di Bari, Bari, Italy; 3 Università degli Studi di Bari Aldo Moro, Dipartimento Interateneo di Fisica, Bari, Italy; 4 Predict S.r.l., Viale Adriatico - Fiera del Levante - Pad. 105, Bari, Italy; 5 Università degli Studi di Bari Aldo Moro, Dipartimento di Farmacia-Scienze del Farmaco, Bari, Italy; 6 Università degli Studi di Bari Aldo Moro, Dipartimento di Scienze del Suolo, della Pianta e degli Alimenti, Bari, Italy; Leiden University, NETHERLANDS, KINGDOM OF THE

## Abstract

University rankings are published on a regular basis and taken as a reference by a widespread audience of students, researchers, and companies. Nonetheless, rankings can be affected by socio-economic dragging effects, since they often fail to incorporate information on the variegated conditions in which scores are reached. This inability to capture structural inequalities can generate self-reinforcing awarding mechanisms, e.g. in performance-based funding distribution, that amplify existing gaps and prevent from recognizing achievements of universities in difficult or emerging contexts. In a previous study, we demonstrated the existence of a socio-economic territorial bias in general rankings, which rate the global performance of institutions. However, the interplay of the variety of territorial contexts and the different features of specific disciplines can give rise to more complex effects. In this work, we investigate the influence of the local socio-economic condition on the performance of universities in rankings, considering a multidimensional representation of the phenomenon, involving the dependence on subject, time, and type of ranking. Our findings show that bibliometric rankings are significantly more affected than reputational ones by socio-economic dragging, which strikingly emerges especially in the natural and life science areas. We conclude the analysis by decoupling territorial dragging effects from the achieved ranked scores. Universities that benefit the most from the mitigation of the socio-economic territorial bias are typically located in territories, mostly outside Western Europe and North America, hosting either a capital or other important cities.

## Introduction

Since the early 1980s, the tendency to rate the academic and research performances of universities all over the world has become widespread, along with its influence on public and private stakeholders [1,2]. Ranking is a powerful tool for a quantitative assessment of performance in many different areas, based on the choice of an appropriate set of indicators. However, drawing up an ordered list of items can have serious drawbacks if academic institutions are the subjects of this analysis. Turning articulated and heterogeneous systems such as universities into an ordered list entails profound over-simplifications [3], which, in particular, tend to hide the context in which they operate. As pointed out by the European University Association [4], “*rankings describe institutional quality according to a very limited set of parameters, which are – by and large – the same for all institutions, independent of their size, location, mission, and financing model, among other factors.*” Furthermore, a favorable socio-economic environment, characterized by effective infrastructures, an active and innovation-oriented industrial system, high employment rate and attractiveness for qualified human capital, can determine a competitive advantage for academic institutions based therein [5–8].

The failure to incorporate information on structural inequalities and territorial diversification among universities represents a problem, especially when rankings become the basis to decide the allocation of public and private funding [9–11]: academic institutions located in more favorable territories tend to achieve better scores and thus are more easily rewarded [12–14], increasing the gap with institutions that have no access to the same advantages [7,15–19].

In a recent study, the existence of *territorial biases*, namely significant dragging effects determined by local socio-economic conditions, was demonstrated for two university rankings, structurally different in their purpose and geographical coverage [20]. The first ranking considered therein, compiled by Times Higher Education, has a general purpose, since it is focused on identifying the best universities on a global scale, evaluating the performance of more than 1000 institutions in terms of education, research, international outlook and technological transfer. The second ranking is compiled by CENSIS and is referred to a restricted geographical and thematic context, since it rates the performances of the Italian universities in terms of student services. Despite their difference, these two rankings share the same approach: they evaluate universities as a whole, based on general criteria, without distinguishing the performances of the composing departments, units dealing with definite fields of learning that, especially in large universities, can be very much diversified from each other.

However, a relevant part of students, researchers and stakeholders that consult rankings are interested in an academic institution comparison that is more focused on specific areas, in which they aim to undertake a course of study, a job, or an investment opportunity [21]. Such a need has determined the rise of several per-subject rankings, in which university performances are referred to a specific learning area [22]. On the other hand, the variety of territorial contexts [14,23,24], the different features of specific disciplines [25,26], and the properties of the indicators employed for rating [27–29], can highlight additional complexity in the phenomenology of territorial biases [7,15,18,30–33].

The research goals of the present article are the (i) detection, (ii) quantification, (iii) comparison and (iv) geographical characterization of territorial biases in rankings referred to different academic subjects, performance metrics (bibliometric or reputational, as detailed in the following), and observation years. For each ranking, we first investigate the presence of a significant socio-economic dragging by evaluating whether or not institutions operating in similar contexts tend to achieve comparable scores. To account for differences inside the same country, we set our geographical resolution to the OECD subregions at Territorial Level 2 (TL2) [34,35]. Then, we compute the *debiased* rankings, in which territorial dragging effects are mitigated, following the procedure defined in [20]. We use the debiased rankings as a benchmark to highlight specific regions where the achievements of universities are significantly underestimated by the original ranking score, that does not properly account for structural inequalities between territories.

Adding more dimensions to the study of structural inequality phenomenology is reasonable in view of the fact that subjects available nowadays in the academic offer are characterized by a diversified degree of intrinsic complexities, and by development trajectories that can be more or less related with the territorial ones. Actually, as observed in Ref. [25], nations achieve different levels of scientific competitiveness in different learning domains; the most complex ones, requiring an advanced and wealthy research system, such as biochemistry and neuroscience, tend to flourish in the most developed nations, while in developing countries the research system is based on more fundamental sciences and on disciplines that are more directly related to a social function, such as engineering and basic medicine. Moreover, biases can be more impactful on a specific category of indicators [27,28,36], and can evolve in time [18], especially considering the abrupt changes in the fields of life sciences induced by the Covid-19 pandemic [37].

To find answers to our research questions, we analyze the per-subject Quacquarelli Symonds (QS) World University Rankings [38,39], for the years from 2020 to 2023. In these rankings, the university score involves both reputational and bibliometric indicators. The reputational ones are acquired through surveys answered by academy and company personalities, to which it is requested to indicate, both on a local and national scale, institutions that they consider excellent for academic performance and graduate students hiring, respectively. The bibliometric indicators are computed from research impact indexes of academic personnel in the learning area of interest. Considering the very different nature of the two types of indicators that concur to the QS rankings, in this work we will also investigate whether a territorial bias acts in diversified ways on either of them.

The most relevant part of the toolbox employed in this analysis is provided by complex networks, a widely used instrument of complex systems physics, already applied to many different fields such as economics [23,40–42], finance [43–45], sustainability [46–48], performance evaluation [49], neuroscience [50–53], human behaviour [54,55], genetics [56–58], also using innovative tools such as multilayer networks [59], higher-order connections among nodes [60], and quantum potentials that encode network connectivity [61,62]. The present work is based on the construction of networks consisting of nodes that coincide with universities, whose mutual connections are weighted according to a given similarity criterion. In particular, we focus on territorial university networks, in which the strength of a link between two institutions is determined by the correlation between the socio-economic indicators of their respective TL2 regions. The presence of a territorial bias is investigated through an assortativity analysis, which quantifies the statistical tendency of nodes (in this case, universities) to connect with other nodes characterized by similar values of a given attribute (in this case, the ranking score). Moreover, we adopt network community detection to cluster universities by similarity into non-overlapping groups, that provide the benchmark to define debiased rankings.

The article is organized as follows. In the “Results” section, we present the findings of our study, focused on a comparison of territorial biases across ranking types, years, subjects and geographical areas. In the “Discussion” section, we interpret the meaning of our findings and present future research perspectives. In the “Materials and Methods” section, we outline the data collection process and the mathematical toolkit of complex network construction and analysis, employed to quantify the ranking biases.

## Results

**Fig 1:**
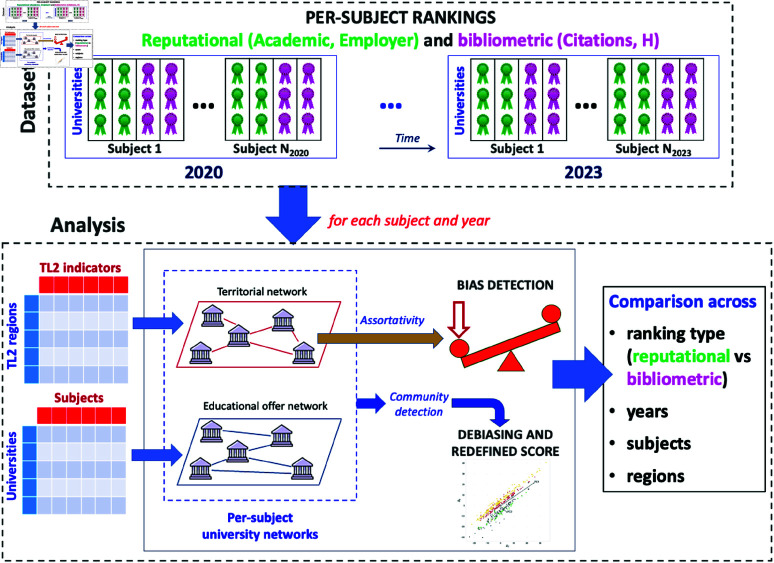
Scheme of the workflow. Territorial biases in per-subject rankings, published in Quacquarelli Symonds World University Rankings for the years from 2020 to 2023, are determined by constructing territorial networks of academic institutions and quantifying their assortativity with respect to the ranked scores. Community detection in the territorial networks and in additional educational offer networks provide the basis to decouple the bias from the ranking, thus obtaining a fairer performance evaluation. The measured territorial biases are compared across ranking types, years, subjects and subregions.

The workflow followed in this study is illustrated in Fig 1. We consider the QS per-subject rankings related to the years from 2020 to 2023, distinguishing the reputational performance metrics (Academic, Employer) from the bibliometric ones (Citations, H). For each ranking, corresponding to a given combination of performance metrics, year, and subject, we construct a territorial university network, whose connections among the ranked academic institutions are determined by the Pearson correlation of OECD indicators referred to the TL2 subregions in which they operate. We then compute the assortativity of such a network with respect to the ranked scores, which quantifies the territorial bias [20].

As detailed in the “Assortativity” subsection of “Materials and Methods”, a given assortativity value *r*_*w*_ can be interpreted as a weighted Pearson correlation, which allows to associate it a with a standard error *S*_*w*_ and a statistical significance, evaluated assuming as a null hypothesis that the standardized assortativity $t=r_{w}/S_{w}$ follows a Student t-distribution. Following this approach, we adopt as a territorial bias quantifier the significance score *s*, a function of the standardized assortativity that is connected to the p-value by the relation *p* = 10^−*s*^ (see “Materials and Methods”). Therefore, using this quantity is convenient to compare assortativity values related to networks of different size, since it determines statistical significance thresholds that are independent of the number of degrees of freedom. In the following, we will consider *r*_*w*_ to be significant provided $10^{-\text{s}}<0.05.$ It is worth noticing that for large values of *t* and large number of degrees of freedom, *s* becomes proportional to the squared standardized assortativity [63].

The assortativity analysis is then used to compare territorial biases across ranking types, years and subjects. To identify the subregions that are most affected by the territorial bias, we include in the framework an educational offer network, which reproduces the similarity among the degree course offers of universities included in the considered ranking. Both kinds of network are then used to define a debiased score through community detection, following the approach of Ref. [20].

### Territorial bias comparison across ranking types

**Fig 2:**
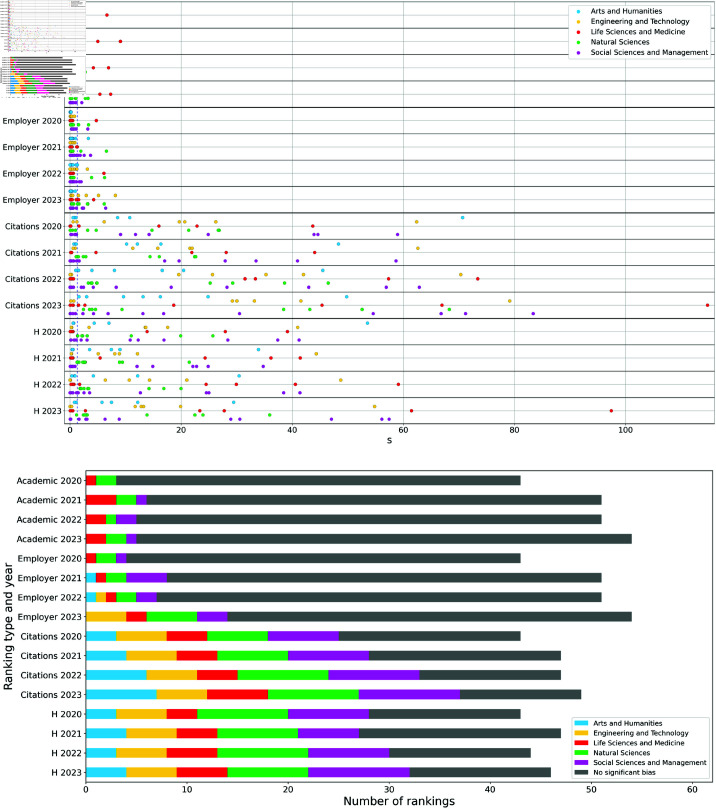
Upper panel. Distribution of statistical significance scores *s* assigned to assortativity of per-subject rankings grouped by type, year and macro-area. The dashed vertical line at s≃1.3 represents the significance threshold. *Lower panel.* Count of per-subject rankings with significant and insignificant assortativity, with the former divided by macro-area.

The distributions of the significance score *s* for all ranking types (Academic, Employer, Citations, H), years (from 2020 to 2023), and subject macro-areas (*Arts and Humanities*, *Engineering and Technology*, *Life Sciences and Medicine*, *Natural Sciences*, *Social Sciences and Management*), are shown in the upper panel of Fig 2. The visual qualitative difference of *s* scores for reputational and bibliometric performance indicators is confirmed by the count of per-subject rankings with significant and insignificant assortativity, with the former grouped by subject macro-areas, reported in the lower panel of Fig 2. To evaluate the statistical significance of the aforementioned differences between ranking types, we compare at each fixed year:

- the distributions of *s* values, using the Wilcoxon ranksums test and the t-test;
- the fractions of subjects reporting a statistically significant territorial bias (i.e. $10^{-\text{s}}<0.05$), using the statistical z-test on proportions.

All these tests return the same evidence: bibliometric rankings are significantly more biased than the reputational ones throughout all the considered timespan, with p-values below 0.05 (see Table S1 in S1 Appendix for a detailed report).

### Territorial bias comparison across years

The assortativity analysis allows to check whether significant time variations in the territorial bias that affects rankings occurred in the period from 2020 to 2023. For each ranking type, we compare across the years the distributions of *s*, using both the Wilcoxon ranksums test and the t-test. Furthermore, we investigate the significance of time changes in the fraction of subjects with a statistically significant territorial bias, by applying the statistical z-test on proportions to compare distributions referred to the same ranking type and different years.

The statistical tests highlight two significant increases in the territorial bias affecting per-subject rankings: one between the Employer rankings in 2020 and 2023, and one between the Citations rankings in 2021 and 2023 (detailed results in the Table S2 in S1 Appendix).

### Territorial bias comparison across subjects

**Fig 3:**
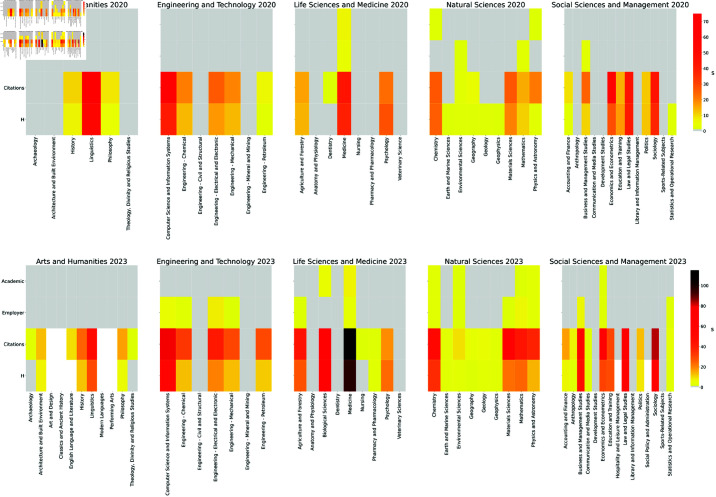
Significance score s quantifying the territorial bias in per-subject rankings for different macro-areas and performance indicators, in 2020 (upper panel) and 2023 (lower panel). Grey cells correspond to statistically insignificant assortativities. Analogous plots referred to 2021 and 2022 are reported in S1 Appendix (Fig. S1).

From an inspection of the upper panel of Fig 2, it is evident that some educational macro-areas tend to be more prone than others to territorial biases, at least in specific rankings. We refine this kind of analysis by investigating if territorial biases act in diversified ways on rankings referred to different disciplines. Fig 3 provides, for the years 2020 and 2023, a visual comparison among the *s* distributions across different ranking types and subject macro-areas. The analogous plots for 2021 and 2022 are reported in S1 Appendix (Fig. S1). The five most biased rankings for each year are listed below.

- **2020**: (1) *Linguistics* – Citations, (2) *Computer Science and Information Systems* – Citations, (3) *Economics and Econometrics* – Citations, (4) *Linguistics* – H, (5) *Law and Legal Studies* – Citations.
- **2021**: (1) *Computer Science and Information Systems* – Citations, (2) *Economics and Econometrics* – Citations, (3) *Linguistics* – Citations, (4) *Computer Science and Information Systems* – H, (5) *Biological Sciences* – Citations.
- **2022**: (1) *Medicine* – Citations, (2) *Computer Science and Information Systems* – Citations, (3) *Economics and Econometrics* – Citations, (4) *Medicine* – H, (5) *Biological Sciences* – Citations.
- **2023**: (1) *Medicine* – Citations, (2) *Medicine* – H, (3) *Sociology* – Citations, (4) *Computer Science and Information Systems* – Citations, (5) *Law and Legal Studies* – Citations.

Besides highlighting the per-subject rankings that are most affected by territorial biases, it is worth investigating how the latter tend to be distributed within subject macro-areas. To address this point, for each set of rankings corresponding to a given combination of type and year, we use the z-test on proportions to compare among macro-areas the share of subjects with statistically significant territorial bias. The outcome of this analysis, presented in detail in S1 Appendix (Table S3), suggests that hierarchies of bias distributions tend to change with the ranking type. The only exception is represented by the set of H rankings within the Natural Science domain, which is characterized by a significantly larger fraction of biased per-subject rankings than the other domains.

### Territorial bias comparison across subregions

While the results described up to now are based on the assortativity analysis, providing an evaluation of the global impact of the territorial bias on rankings, to investigate the effect of such a bias across subregions requires focusing on scores of each single university. In such a way, one can determine whether institutions of specific subregions are more significantly affected by the bias than others.

For this purpose, we apply to significantly biased rankings ($10^{-\text{s}}<0.05$) a similar pipeline as the one used in Ref. [20] to determine the bias on the ranking outcomes of each university. Technical details of the whole procedure are presented in the “Methods” section. First, given the ranking, we construct an additional network, in which a pair of universities can be connected with a link whose weight is determined by the overlap of their educational offer. Then, a community detection procedure is performed on both the territorial and the educational offer networks, providing unsupervised optimal partitions based on the two different kinds of similarity. We remark that, based on the results of Ref. [20], no relevant bias related to the educational offer is expected. For a given ranking, the network communities provide the basis to define, for each ranked university, the *debiasing parameters* $\delta_{T}$ and $\delta_{E}$, computed as the difference between its ranked score and a weighted average of those achieved by its community peers in the territorial and educational offer network, respectively. Finally, to mitigate the bias, we perform principal component analysis in the ($\delta_{T},\delta_{E}$) plane. We then determine the principal component PC1 for which territorial network assortativity is smaller, which is labeled as the *debiased score*.

**Fig 4:**
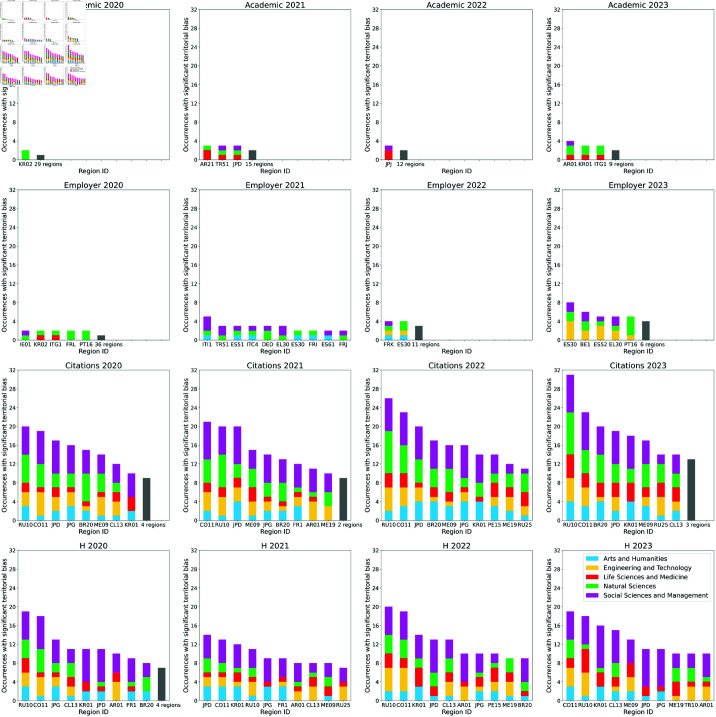
Top 10 subregions by occurrence among the ones with a significant position improvement of their universities in PC1 with respect to the original ranking, for different performance metrics and years. The labels on the horizontal axes are referred to the official TL2 subregion IDs provided by OECD [34,35], with the first two letters identifying country.

The described framework can be used to evaluate how the territorial bias differently affects subregions, through the comparison between debiased PC1 rankings and their original counterparts. In a given ranking, we compute for each university *u* the difference Δrank(u) between the positions achieved in terms of debiased and original scores. Then, we group such Δrank values based on the subregions in which universities operate. For each given region, we consider the universities $u_{1},\ldots ,u_{N}$ located therein, and perform a statistical comparison between the distribution of values $\left\{\Delta rank\left(u_{1}\right),\ldots ,\Delta rank\left(u_{N}\right)\right\}$ and the overall distribution of Δrank for universities in all the other subregions. This comparison is made through the one-way Wilcoxon ranksums test with a 5% significance threshold. The described procedure highlights the subregions for which the position increase in a given PC1 debiased ranking is statistically significant. In Fig 4, we report for each ranking type and for each year the ten subregions that appear most frequently among those with a significant improvement in PC1 with respect to the original ranking. Universities in these subregions thus tend to perform significantly better than the expectation based on their community membership, in a large number of cases.

It is evident from Fig 4 that the position gains are much more relevant for the bibliometric rankings than for the reputational ones, following the tendency of stronger bias affecting the former observed in Fig 2. In the bibliometric rankings, we find recurring cases of TL2 subregions outside Western Europe and North America, often hosting the capital city (AR01 Buenos Aires – Argentina, CO11 Bogotá Capital District – Colombia, CL13 Santiago – Chile, JPD Southern Kanto – Japan, KR01 Capital Region – Korea, ME09 Distrito Federal – Mexico, PE15 Lima – Peru, RU10 Moscow Oblast – Russian Federation) or, less frequently, other outstandingly relevant cities (BR20 S ao Paulo – Brazil, JPG Kansai – Japan, ME19 Nuevo León – Mexico, RU25 Leningrad Oblast – Russian Federation). The only exception in Western Europe is represented by the French capital region FR1 Île-de-France – France. It is worth remarking that the occurrences of a statistically significant improvement for the aforementioned subregions tend to be uniform across disciplinary areas. In the case of the reputational rankings, where occurrences of significant position increases are much less, TL2 subregions of Southern Europe (Spain, south of France, Italy, Greece) are highly represented, with other cases pertaining to Argentina, Belgium, Germany, Ireland, Japan, Korea, and Turkey.

## Discussion

The main purpose of university rankings is to highlight the best performing academic institutions according to some indicators. Nevertheless, as shown in previous studies [12–14,20,64–66], territorial context factors affect university performances. In this study, we focused on detecting territorial biases, quantified through network assortativity, in per-subject rankings. The analysis reveals that this socio-economic dragging effect is much more significant in the bibliometric rankings (Citations and H) than in the reputational ones (Academic and Employer). Such a difference is motivated by the procedure that defines both reputational scores: as detailed in Materials and Methods, they are based on surveys addressed to university (Academic) and industry (Employer) actors, who are requested to indicate 10 national and 30 international institutions that they consider excellent for research activity and graduate recruitment, respectively. Therefore, the coexistence of the global and local geographical scales in the survey answers acts as a mitigator for the socio-economic dragging. On the other hand, the remarkable territorial biases observed in the Citations and H rankings corroborate independent results reported in the literature [67], which relate quantitative performance in bibliometric indicators to external factors such as greater funding availability [25,68] and access to cutting-edge research facilities [6], or institutional prestige and author reputation [69]. These results suggest that mitigating the dragging effect in bibliometric rankings, as we did through debiasing, can lead to a fairer comparison of the activities performed by academic institutions operating in variegated territorial contexts.

Focusing on subjects, one can notice the multiple occurrence of specific disciplines in the lists of five most biased rankings per year reported in the “Results” section: *Computer Science and Information Systems* (5 times), *Medicine* (4, in the last 2 years), *Linguistics* (3, in the first 2 years), *Economics and Econometrics* (3), *Law and Legal Studies* (2), *Biological Sciences* (2). A territorial bias in *Medicine* and *Biological Sciences* can be explained by the fact that these subjects rely on the collection of data through practices such as patient recruitment, clinical trials, preservation and analysis of lab samples, which can be heavily influenced by territorial assets [70], including the availability of cutting-edge laboratories and infrastructures [71,72], and the presence of efficient hospitals [73]. On the other hand, the statistical significance of territorial biases for *Medicine* (especially) and *Biological Sciences* shows an increase with time, which could have a correlation with the expanding amount of literature on Covid-19 [37]. Territorial wealth impacts on *Computer Science and Information Systems* bibliometric rankings, which appear among the 5 most biased ones for all years, in diversified ways. Firstly, the presence in developed countries of an increasing number of infrastructures for high-performance computing represents a relevant edge for researchers in the field [71]. Moreover, in the *Computer Science and Information Systems* research field, papers presented at conferences are regularly peer-reviewed, considered full-fledged research articles and typically published in high-impact journals [36]. Of course, access to conferences, and thus to these opportunities for publication in renowned journals, is subject to the availability of funds, which are distributed in an uneven way among institutions. The bias in *Linguistics* can be explained by the non-homogeneity of production and impact of research in different languages [74]. Such a point of view is corroborated by the fact that the universities with the largest scores in the *Linguistics* – Citations rankings are all based in English-speaking countries. We can infer that the reasons behind the bias in the *Economics and Econometrics* and *Law and Legal Studies* sectors are due to a similar intrinsic bias, related to the fact that scientific production in these fields is focused on specific social and economic contexts, which are essentially those in which the most important academic institutions are embedded [75,76]. Concerning the disciplinary macro-areas, biases tend to be evenly distributed among them, with alternating hierarchies. A notable exception is represented by *Natural Sciences*, a sector in which research is notoriously focused on bibliometrics, whose share of subjects with a biased H ranking is significantly larger than for other macro-areas. It is worth remarking that such a finding corroborates a problematic view on the use of the H index for the evaluation of scientific production, considering the statistical irregularity of citation distributions [77].

The last part of our study focused on a possible geographical characterization of the socio-economic dragging effects in rankings. Community detection in the territorial and educational offer university networks provided the basis to identify academic institutions that perform better (in a statistically significant way) than expected from the results achieved by their community peers. The complex network approach presented in our work, based on evaluating the debiasing parameters and the principal components of their distribution, allows to quantify and partially overcome such a bias, thus introducing a fairer rating scheme. The results of the geographical analysis are especially interesting for the bibliometric scores, where debiasing generates a larger number of position changes in the ranking, compared to the reputational scores. In this case, it emerges that the subregions where universities are most penalized by the territorial bias typically host the capital or one of the outstandingly relevant cities of countries outside North America and Western Europe (with the notable exception of Île-de-France). Universities based therein typically perform better than their community peers, and thus achieve the largest position improvements in the debiasing procedure. This result can be ascribed to higher-education systems in which excellence centers are concentrated in a small number of cities. Most cases refer to countries that face objective challenges such as high inequalities, brain drain, large distance between academic centers, or a combination of these factors. On the other hand, it is worth noticing that the significant position improvement of subregions in highly developed non-Western countries such as Japan and Korea may also be an indicator of the gap they accumulate in the original bibliometric ranking for being partially out of Western “citation clubs” [33].

We finally remark that the objective of our analysis is not so much to criticize bibliometric rankings, which have the advantage of being based on measurable data, but to suggest a careful interpretation of them and possibly a mechanism of wealth-dragging mitigation, especially in the context of third-party evaluations. The problem with the questionable fairness of scores is clearly acknowledged in the CoARA “Agreement on reforming research assessment” (2022) [78], which warns against the use of research organization rankings in the assessment of both institutions and individuals. Furthermore, the oversimplified (and sometimes intrinsically flawed) representation of the academic world provided by rankings is pointed out by the European University Association in the document “Key considerations for the use of rankings by higher education institutions” (2023) [4], already quoted in the Introduction.

Future research on the topic will be oriented to a complementary research question: determining how much specific advantageous features of a territory, especially related to social and economic development, are correlated to the presence of universities that provide outstanding performances in specific subjects. On the other hand, a possible generalization of the present research would be obtained by considering, besides quantitative socio-economic indicators, also subjective ones such as the perceived quality of local or regional governments [79]. Moreover, a more robust analysis of the bias variation in time would be possible when more years of observation of the QS rankings will be available.

## Materials and methods

### Rankings

In this work, we analyze the per-subject Quacquarelli Symonds (QS) World University Rankings [38,39], which measure university performance based on four indicators [80]:

- **Academic.** This index evaluates the responses of about 130,000 academic personalities. Respondents are requested to indicate their area of expertise and to list up to 10 national and 30 international institutions that they consider excellent in terms of research in the given area.
- **Employer.** This index is based on the survey responses of more than 75,000 graduate employers worldwide, who are asked to identify the disciplines from which they prefer to recruit, and to name up to 10 national and 30 international institutions that they consider excellent for the recruitment of graduates.
- **Citations.** This index measures the number of citations per paper authored by faculty members of each university in a given sector. Data are retrieved from the Elsevier Scopus database [81]. A minimum publication threshold is set for each subject to avoid anomalies due to small numbers of highly cited papers. This threshold is adapted in view of reflecting prevalent publication and citation patterns for a given subject.
- **H.** The H index measures both the productivity and impact of single academic personalities or departments. The value of the index represents the highest number of authored papers with at least the same number of citations.

In addition to the previous specialized indicators, rankings also feature an overall *Score*, which we neglect since it is partially filled only for universities in the top ranking positions.

We consider the QS per-subject rankings for the years 2020 (involving 43 subjects), 2021 (51), 2022 (51), and 2023 (54). For each year, subjects are included in five macro-areas: *Arts and Humanities*, *Engineering and Technology*, *Life Sciences and Medicine*, *Natural Sciences*, and *Social Sciences and Management*. The membership of subjects to macro-areas is reported in S1 Appendix (Table S4). In 2023, three new subjects appeared (*Art History*, *Data Science*, *Marketing*), that were still not formally framed into any macro-area. Rankings related to these subjects are included in the analysis, but do not contribute to considerations related to the educational macro-areas. Notice that the yearly bibliometric rankings are not available for all subjects, as reported in S1 Appendix (Table S4).

The list of the 1237 universities appearing in at least one ranking in the considered years, and located in TL2 subregions for which socio-economic indicators are available, is reported in S1 File. The lists of universities for each specific per-subject ranking are reported in S2 File (year 2020), S3 File (year 2021), S4 File (year 2022), S5 File (year 2023).

### Complex network construction

In this section, we describe the procedure to build the two kinds of complex network on which our results are based: the territorial network, constructed from the socio-economic similarity of different OECD territories where universities are established, and the educational offer network, determined by overlap in the lists of subjects available at the different institutions. The whole network construction process is repeated for each subject and each year, as illustrated in Fig 1.

#### Territorial network

Territorial indicators are collected from OECD Regional Statistical Dataset [34]. In particular, we set our resolution to the OECD subregions at Territorial Level 2 (TL2) [35], with the only exceptions of Estonia and Latvia, for which Territorial Level 3 (TL3) subregions are considered, mainly for reasons of data availability. It should be noticed that some data missing from the OECD archives, namely infant mortality in Brazil and life expectancy at birth in Argentina and Brazil, are collected from Global Data Lab [82].

The data collection procedure initially returns 299 territorial indicators, available for 286 subregions. To avoid redundancy, we discard indicators whose mutual Pearson correlation with at least another one is larger than 0.9: specifically, if two indicators are strongly correlated with each other, we retain the one with more available values. Furthermore, we verify that no indicator is characterized by vanishing standard deviation. In order to mitigate the effect of outliers, the values above the 99th percentile and those under the 1st percentile of each indicator are replaced by the reference percentiles. Finally, indices are normalized between 0 and 1, to obtain equally-scaled data. The described preprocessing operations provide a dataset of 241 territorial indicators (listed in S6 File), pertaining to 286 TL2 subregions.

For each ranking, corresponding to a given combination of performance metrics, year and subject, we first construct a *subregion network* in which nodes, corresponding to the OECD subregions where ranked universities are based, are connected if the Pearson correlation between the sets of their socio-economic indicators is statistically significant (*p*<0.01). The robustness of the obtained results against variations of such a significance threshold is proved by the results reported in S1 Appendix (Table S5). The value of the Pearson correlation is then assigned as a weight to the existing links. Then, we use the subregion network to construct a *territorial network of universities*, in which nodes coincide with academic institutions reported in the considered ranking: if two or more nodes belong to the same region, they are connected with link weight 1; otherwise, the connection depends on the presence of a link in the subregion network, also inheriting the link weight thereof.

#### Educational offer network

For each year, we construct a matrix with rows corresponding to all the academic institutions involved in at least one ranking for that year, and columns corresponding to subjects. The element of this matrix associated with university *u* and subject *subj* is equal to 1 if *u* appears in any ranking of *subj* in the year of interest, and 0 otherwise. The dimensions of the matrix are determined, for each year, by the number of universities and subjects involved in the rankings:

- 1303 universities and 43 subjects in 2020,
- 1621 universities and 51 subjects in 2021,
- 1557 universities and 51 subjects in 2022,
- 1605 universities and 54 subjects in 2023.

For each ranking (namely, for a given combination of year, performance metrics, and subject), we consider the matrix of the related year and select only the rows corresponding to the universities featured in the ranking. Such a sub-matrix is used to construct the educational offer network for that ranking.

In the educational offer network, nodes correspond to ranked universities, and the weight of the possible link between institutions *u* and *v* is determined by the Dice index

$$DSC_{uv}=\frac{2|\Gamma_{u}\cap \Gamma_{v}|}{\left|\Gamma_{u}|+|\Gamma_{v}\right|},$$ (1)

where $\Gamma_{u}$ and $\Gamma_{v}$ are the sets of subjects for which universities *u* and *v* appear in the respective rankings, and |…| denotes the set cardinality. Such a procedure would in principle provide a complete network.

To obtain an educational offer network ℰ that is neither too dense nor too sparse, we start from a network ${ℰ}_{0}$ where ranked universities are connected with a weight determined by Eq. (1). Then, we extract from ${ℰ}_{0}$ its maximum-spanning tree ${ℰ}_{1}$ [83]. Afterwards, we gradually add to ${ℰ}_{1}$ the maximum-weight links of ${ℰ}_{0}$. The process stops, providing the final network ℰ, when the link density is closest to that of the positive-weight links in the corresponding territorial network. The robustness of the obtained results against variations of the density or inclusion of all the links is shown in S1 Appendix (Table S6).

### Assortativity

In an arbitrary network, one can assign attributes to nodes, in order to enrich the description of the system under investigation. If a numerical attribute is assigned, one can quantify the tendency of nodes with similar attribute values to be connected with each other through the *assortativity* [84], defined for a binary network as

$$r=\frac{\sum_{ij}(A_{ij}-\frac{k_{i}k_{j}}{2m})x_{i}x_{j}}{\sum_{ij}(k_{i}\delta_{ij}-\frac{k_{i}k_{j}}{2m})x_{i}x_{j}},$$ (2)

where *x*_*i*_ is the attribute value of node *i*, *k*_*i*_ is the degree of node *i*, *A*_*ij*_ is an element of the adjacency matrix, and *m* is the total number of edges in the network. The assortativity has a value in the interval [–1,1], where –1 indicates the tendency to connect nodes with very different attribute values, while 1 corresponds to the case where links connect only nodes with the same attribute value. In the intermediate case *r* = 0, there is no relevant linear correlation between the attributes of nodes connected by edges.

The previous definition can be generalized to weighted networks [85],

$$r_{w}=\frac{\sum_{ij}(w_{ij}-\frac{s_{i}s_{j}}{W})x_{i}x_{j}}{\sum_{ij}(s_{j}\delta_{ij}-\frac{s_{i}s_{j}}{W})x_{i}x_{j}},$$ (3)

where *w*_*ij*_ is the weight of the link between node *i* and node *j*, $s_{j}=\sum_{i}w_{ij}$ is the strength of node *j*, and $W=\sum_{ij}w_{ij}$. The above definition is meaningful only if weights are non-negative. Therefore, since the territorial networks contain links with both positive and negative weights, we replace each network with the corresponding sub-network containing only positive-weight links. This does not entail a relevant loss of information, since the fraction of negative-weight links, averaged over subjects, is smaller than 3.5% for all the considered years.

For a weighted network, the assortativity formally corresponds to the weighted Pearson correlation between two vectors having both length 2*m*, with *m* the total number of links, whose values are the attributes *x*_*i*_ and *x*_*j*_ of each connected node pair (*i*,*j*); the contribution of $\left(x_{i},x_{j}\right)$ to the overall correlation is weighted by *w*_*ij*_.

The statistical significance of a given assortativity value *r*_*w*_ can be evaluated, based on the identification with a weighted Pearson correlation, by associating a standard error

$$S_{w}=\sqrt{\frac{1-r_{w}^{2}}{2m-2}}$$ (4)

and assuming as a null hypothesis that the variable $t=r_{w}/S_{w}$ follows a Student t-distribution

$$f_{\nu }(t)=C_{\nu }{\left(1+\frac{t^{2}}{\nu }\right)}^{-\frac{\nu +1}{2}},$$ (5)

with ν=2m degrees of freedom and $C_{\nu }$ a normalization coefficient [63]. The significance score

$$s(t)=-\log_{10}\left(2\int_{\left|t\right|}^{\infty }f_{\nu }(t^{\prime })dt^{\prime }\right),$$ (6)

defined in a way that the p-value can be obtained as $10^{-\text{s}}$, becomes asymptotically proportional to ${\text{t}}^{2}$ for large values of *t* and large numbers of degrees of freedom, when $f_{\nu }(t)$ approaches a standard normal distribution. Assortativity, standard error and significance scores are computed through the ‘weights’ R library [86].

### Community detection

Community detection is performed using the Leiden algorithm [87,88], with the resolution γ treated as a free parameter, varying in [0.5,1] with a 0.05 step, and the remaining algorithm parameters fixed to default (β=0.05, objective function = modularity). For each value of resolution, *K* = 100 algorithm runs are performed, each with a different pseudorandom number generator seed; we use majority voting to choose among the resulting partitions. The procedure is made more robust by using a stability criterion, that considers the similarity of different partitions $\{p_{j}{\}}_{\left(j=1,..,K\right)}$, based on the average Normalized Mutual Information

$$\left\langle NMI\right\rangle =\frac{2}{K(K-1)}\sum_{a=1}^{K-1}\sum_{b=a+1}^{K}NMI(p_{a},p_{b}),$$ (7)

where $NMI(p_{a},p_{b})$ is the Normalized Mutual Information between a given pair of partitions, and K(K−1)/2 is the number of distinct pairs. The majority partition over *K* = 100 runs can be approved only if ⟨NMI⟩≥0.95, and if

- it is non-trivial (i.e., not consisting of a single community);
- it is not too fragmented, namely it does not contain communities whose cardinality is less than 5% of the cardinality of the partitioned network.

If the majority voting results obtained for 100 runs, at different values of the resolution γ, satisfy the above conditions, we choose the output with larger ⟨NMI⟩, and the majority partition corresponding to this choice is identified as the result of community detection. The procedure is applied in a hierarchical algorithm, in which at each step the communities obtained at the previous step are partitioned according to the same criteria. The hierarchical community detection stops when no partition obtained at a given step satisfies the stability, non-triviality and non-fragmentation criteria.

### Debiased score

Community membership represents the starting point to define a fair performance evaluation. For each combination of year, performance metrics, and subject, given the ranking *I* we associate to each university *u* the debiasing parameters $\delta_{T}(u)$ and $\delta_{E}(u)$, defined as

$$\delta_{S}(u)=I(u)-\frac{\sum_{v\in C_{S}}w_{uv}^{S}I(v)}{\sum_{v\in C_{S}}w_{uv}^{S}},\;\text{with }S=T,E$$ (8)

where *C*_*T*_ and *C*_*E*_ are the territorial and educational offer communities to which *u* belongs, and *I*(*u*) is the ranked score achieved by *u*. The subtracted quantity is the average of the score *I* in the rest of the community, weighted by the (*u*,*v*) edge weights $w_{uv}^{S},\;(S=T,E)$ of the considered network. In this way, community peers that are weakly connected to *u* give a negligible contribution to Eq. (8). If the debiasing parameter is positive (negative), the performance of *u* is better (worse) than the one expected, based on community membership. As an example, we report in S1 Appendix (Fig. S2) the scatter plots of $\left(\delta_{T},\delta_{E}\right)$ values referred to the Citations ranking of the subject *Business and Management Studies* for the year 2020, along with the network on which the detection of territorial communities is based.

If the territorial network is significantly assortative with respect to the considered ranking, one can observe in the distribution of $\left(\delta_{T},\delta_{E}\right)$ values a grouping in terms of territorial communities, mostly occurring along a direction that is orthogonal to the one of maximal variance. Therefore, to quantify such a tendency and mitigate the territorial bias in the ranking, we determine the principal components of the $\left(\delta_{T},\delta_{E}\right)$ distributions. The higher-variance principal component PC1, which turns out to be the less assortative with respect to the territorial network, constitutes a redefined (debiased) ranking, in which geographical influence is mitigated; the remaining principal component PC2, instead, provides a measure of the territorial dragging effect on the original scores [20].

## Supporting information

S1 File Full list of universities. List of the 1237 universities appearing in at least one per-subject ranking from 2020 to 2023, and located in the TL2 subregions for which socio-economic indicators are available.
(XLSX)

S2 File List of universities in 2020 rankings. Database of 43 sheets, each containing the list of universities appearing in a specific 2020 per-subject ranking, located in the TL2 subregions for which socio-economic indicators are available.
(XLSX)

S3 File List of universities in 2021 rankings. Database of 51 sheets, each containing the list of universities appearing in a specific 2021 per-subject ranking, located in the TL2 subregions for which socio-economic indicators are available.
(XLSX)

S4 File List of universities in 2022 rankings. Database of 51 sheets, each containing the list of universities appearing in a specific 2022 per-subject ranking, located in the TL2 subregions for which socio-economic indicators are available.
(XLSX)

S5 File List of universities in 2023 rankings. Database of 54 sheets, each containing the list of universities appearing in a specific 2023 per-subject ranking, located in the TL2 subregions for which socio-economic indicators are available.
(XLSX)

S6 File List of socio-economic indicators. List of the 241 socio-economic indicators, pertaining to 286 OECD TL2 subregions, used to construct the region networks and the territorial networks of universities.
(XLSX)

S1 Appendix Supporting tables and figures. We report in this Appendix six tables and two figures that support the findings presented in the main text.
(PDF)
