# Supplementary material for: Network assortativity for a multidimensional evaluation of socio-economic territorial biases in university rankings
Source: PLoS One. 2025 Jun 10;20(6):e0323356. doi: 10.1371/journal.pone.0323356 (PMC12151419; doi:10.1371/journal.pone.0323356)
Supplement: S1 Appendix — We report in this Appendix six tables and two figures that support the findings presented in the main text. (PDF) [file pone.0323356.s007.pdf]

S1 APPENDIX
Network assortativity for a multidimensional evaluation of socio-economic territorial biases in university rankings

Loredana Bellantuono[1,2], Andrea Lo Sasso[2,3,4], Nicola Amoroso[2,5*], Alfonso Monaco[2,3], Sabina Tangaro[2,6], Roberto Bellotti[2,3]

1 Università degli Studi di Bari Aldo Moro, Dipartimento di Biomedicina Traslazionale e Neuroscienze (DiBraiN), Bari, I-70124, Italy
2 Istituto Nazionale di Fisica Nucleare, Sezione di Bari, Bari, I-70125, Italy
3 Università degli Studi di Bari Aldo Moro, Dipartimento Interateneo di Fisica, Bari, I-70125, Italy
4 Predict S.r.l., Viale Adriatico - Fiera del Levante - Pad. 105, Bari, I-70132, Italy
5 Università degli Studi di Bari Aldo Moro, Dipartimento di Farmacia-Scienze del Farmaco, Bari, I-70125, Italy
6 Università degli Studi di Bari Aldo Moro, Dipartimento di Scienze del Suolo, della Pianta e degli Alimenti, Bari, I-70126, Italy

* nicola.amoroso@uniba.it

Abstract

We report in this Appendix six tables and two figures that support the findings presented in the main text.

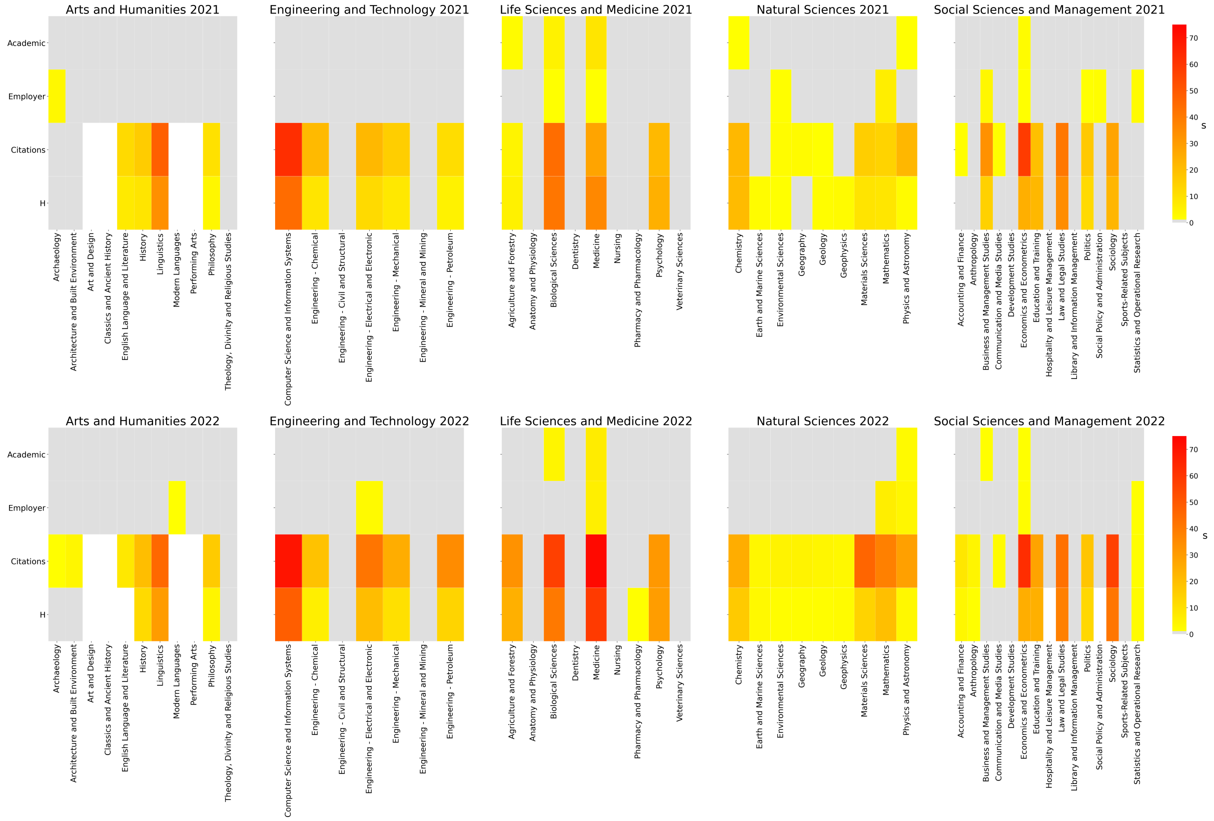

Fig S1. Significance score s quantifying the territorial bias in per-subject rankings for different macro-areas and performance indicators, in 2021 (*upper panel*) and 2022 (*lower panel*). Grey cells correspond to statistically insignificant assortativities.

Year 2020, Wilcoxon rank-sum	**Employer**	**Citations**	**H**
**Academic**	1.67 ($p = 0.0473$)	-4.96 ($p = 3.49 \times 10^{-7}$)	-5.15 ($p = 1.29 \times 10^{-7}$)
**Employer**		-5.33 ($p = 4.82 \times 10^{-8}$)	-5.71 ($p = 5.54 \times 10^{-9}$)
**Citations**			($p > 0.05$)

Year 2020, t-test	**Employer**	**Citations**	**H**
**Academic**	($p > 0.05$)	-4.84 ($p = 2.89 \times 10^{-6}$)	-4.66 ($p = 5.99 \times 10^{-6}$)
**Employer**		-4.87 ($p = 2.59 \times 10^{-6}$)	-4.69 ($p = 5.20 \times 10^{-6}$)
**Citations**			($p > 0.05$)

Year 2020, z-test on proportions	**Employer**	**Citations**	**H**
**Academic**	($p > 0.05$)	-5.06 ($p = 2.07 \times 10^{-7}$)	-5.61 ($p = 9.84 \times 10^{-9}$)
**Employer**		-4.79 ($p = 8.34 \times 10^{-7}$)	-5.35 ($p = 4.30 \times 10^{-8}$)
**Citations**			($p > 0.05$)

Year 2021, Wilcoxon rank-sum	**Employer**	**Citations**	**H**
**Academic**	($p > 0.05$)	-5.21 ($p = 9.50 \times 10^{-8}$)	-4.46 ($p = 4.19 \times 10^{-6}$)
**Employer**		-5.19 ($p = 1.07 \times 10^{-7}$)	-4.68 ($p = 1.47 \times 10^{-6}$)
**Citations**			($p > 0.05$)

Year 2021, t-test	**Employer**	**Citations**	**H**
**Academic**	($p > 0.05$)	-5.28 ($p = 3.98 \times 10^{-7}$)	-4.77 ($p = 3.24 \times 10^{-6}$)
**Employer**		-5.30 ($p = 3.62 \times 10^{-7}$)	-4.80 ($p = 2.86 \times 10^{-6}$)
**Citations**			($p > 0.05$)

Year 2021, z-test on proportions	**Employer**	**Citations**	**H**
**Academic**	($p > 0.05$)	-4.97 ($p = 3.39 \times 10^{-7}$)	-4.78 ($p = 8.74 \times 10^{-7}$)
**Employer**		-4.50 ($p = 3.36 \times 10^{-6}$)	-4.31 ($p = 8.15 \times 10^{-6}$)
**Citations**			($p > 0.05$)

Year 2022, Wilcoxon rank-sum	**Employer**	**Citations**	**H**
**Academic**	($p > 0.05$)	-5.29 ($p = 5.98 \times 10^{-8}$)	-5.46 ($p = 2.34 \times 10^{-8}$)
**Employer**		-5.12 ($p = 1.50 \times 10^{-7}$)	-5.18 ($p = 1.11 \times 10^{-7}$)
**Citations**			($p > 0.05$)

Year 2022, t-test	**Employer**	**Citations**	**H**
**Academic**	($p > 0.05$)	-5.89 ($p = 2.87 \times 10^{-8}$)	-5.23 ($p = 5.17 \times 10^{-7}$)
**Employer**		-5.83 ($p = 3.64 \times 10^{-8}$)	-5.15 ($p = 7.15 \times 10^{-7}$)
**Citations**			1.68 ($p = 0.0481$)

Year 2022, z-test on proportions	**Employer**	**Citations**	**H**
**Academic**	($p > 0.05$)	-6.13 ($p = 4.35 \times 10^{-10}$)	-5.58 ($p = 2.03 \times 10^{-9}$)
**Employer**		-5.68 ($p = 6.58 \times 10^{-9}$)	-5.43 ($p = 2.86 \times 10^{-8}$)
**Citations**			($p > 0.05$)

Year 2023, Wilcoxon rank-sum	**Employer**	**Citations**	**H**
**Academic**	($p > 0.05$)	-6.19 ($p = 2.93 \times 10^{-8}$)	-5.57 ($p = 1.29 \times 10^{-8}$)
**Employer**		-5.64 ($p = 8.53 \times 10^{-9}$)	-4.94 ($p = 3.81 \times 10^{-7}$)
**Citations**			($p > 0.05$)

Year 2023, t-test	**Employer**	**Citations**	**H**
**Academic**	($p > 0.05$)	-5.94 ($p = 2.07 \times 10^{-8}$)	-5.19 ($p = 5.63 \times 10^{-7}$)
**Employer**		-5.81 ($p = 3.58 \times 10^{-8}$)	-5.03 ($p = 1.11 \times 10^{-6}$)
**Citations**			($p > 0.05$)

Year 2023, z-test on prop.	**Employer**	**Citations**	**H**
**Academic**	-2.27 ($p = 0.0115$)	-6.83 ($p = 4.16 \times 10^{-12}$)	-6.23 ($p = 2.40 \times 10^{-10}$)
**Employer**		-5.03 ($p = 2.50 \times 10^{-7}$)	-4.36 ($p = 6.39 \times 10^{-6}$)
**Citations**			($p > 0.05$)

Table S1. Statistical tests to evaluate differences between the distributions of significance score s, comparing two ranking types for the same year. For all the significant test outcomes, the statistical parameter is reported along with the p-value. In all the significant Wilcoxon rank-sum test and t-test outcomes, except the ones involving (Academic,Employer) for 2020 and (Citations,H) for 2022, the alternative hypothesis is that the distribution is centered on smaller values for the ranking type on the rows. In all the significant z-test outcomes, the alternative hypothesis is that the fraction of subjects reporting a statistically significant territorial bias ($10^{-s} < 0.05$) is smaller for the ranking type on the rows.

Academic, Wilcoxon rank-sum	**2021**	**2022**	**2023**
**2020**	$(p > 0.05)$	$(p > 0.05)$	$(p > 0.05)$
**2021**		$(p > 0.05)$	$(p > 0.05)$
**2022**			$(p > 0.05)$

Academic, t-test	**2021**	**2022**	**2023**
**2020**	$(p > 0.05)$	$(p > 0.05)$	$(p > 0.05)$
**2021**		$(p > 0.05)$	$(p > 0.05)$
**2022**			$(p > 0.05)$

Academic, z-test on proportions	**2021**	**2022**	**2023**
**2020**	$(p > 0.05)$	$(p > 0.05)$	$(p > 0.05)$
**2021**		$(p > 0.05)$	$(p > 0.05)$
**2022**			$(p > 0.05)$

Employer, Wilcoxon rank-sum	**2021**	**2022**	**2023**
**2020**	$(p > 0.05)$	$(p > 0.05)$	-1.98 $(p = 0.0237)$
**2021**		$(p > 0.05)$	$(p > 0.05)$
**2022**			$(p > 0.05)$

Employer, t-test	**2021**	**2022**	**2023**
**2020**	$(p > 0.05)$	$(p > 0.05)$	-2.03 $(p = 0.0225)$
**2021**		$(p > 0.05)$	$(p > 0.05)$
**2022**			$(p > 0.05)$

Employer, z-test on proportions	**2021**	**2022**	**2023**
**2020**	$(p > 0.05)$	$(p > 0.05)$	-2.09 $(p = 0.0182)$
**2021**		$(p > 0.05)$	$(p > 0.05)$
**2022**			$(p > 0.05)$

Citations, Wilcoxon rank-sum	**2021**	**2022**	**2023**
**2020**	$(p > 0.05)$	$(p > 0.05)$	$(p > 0.05)$
**2021**		$(p > 0.05)$	-1.67 $(p = 0.0477)$
**2022**			$(p > 0.05)$

Citations, t-test	**2021**	**2022**	**2023**
**2020**	$(p > 0.05)$	$(p > 0.05)$	-1.80 $(p = 0.0375)$
**2021**		$(p > 0.05)$	-2.24 $(p = 0.0138)$
**2022**			$(p > 0.05)$

Citations, z-test on proportions	**2021**	**2022**	**2023**
**2020**	$(p > 0.05)$	$(p > 0.05)$	-1.77 $(p = 0.0380)$
**2021**		$(p > 0.05)$	-1.67 $(p = 0.0475)$
**2022**			$(p > 0.05)$

H, Wilcoxon rank-sum	**2021**	**2022**	**2023**
**2020**	$(p > 0.05)$	$(p > 0.05)$	$(p > 0.05)$
**2021**		$(p > 0.05)$	$(p > 0.05)$
**2022**			$(p > 0.05)$

H, t-test	**2021**	**2022**	**2023**
**2020**	$(p > 0.05)$	$(p > 0.05)$	$(p > 0.05)$
**2021**		$(p > 0.05)$	-1.87 $(p = 0.0320)$
**2022**			$(p > 0.05)$

H, z-test on proportions	**2021**	**2022**	**2023**
**2020**	$(p > 0.05)$	$(p > 0.05)$	$(p > 0.05)$
**2021**		$(p > 0.05)$	$(p > 0.05)$
**2022**			$(p > 0.05)$

Table S2. Statistical tests to evaluate differences between the distributions of significance score s, comparing two years for the ranking type. For all the significant test outcomes, the statistical parameter is reported along with the p-value. In all the significant Wilcoxon rank-sum test and t-test outcomes, the alternative hypothesis is that the distribution is centered on smaller values for the year on the rows. In all the significant z-test outcomes, the alternative hypothesis is that the fraction of subjects reporting a statistically significant territorial bias ($10^{-s} < 0.05$) is smaller for the year on the rows.

Academic 2020	**ET**	**LSM**	**NS**	**SSM**
**AH**	$(p > 0.05)$	$(p > 0.05)$	$(p > 0.05)$	$(p > 0.05)$
**ET**		$(p > 0.05)$	$(p > 0.05)$	$(p > 0.05)$
**LSM**			$(p > 0.05)$	$(p > 0.05)$
**NS**				1.78 $(p = 0.0373)$

H 2020	**ET**	**LSM**	**NS**	**SSM**
**AH**	$(p > 0.05)$	$(p > 0.05)$	-2.37 $(p = 8.85 \times 10^{-3})$	$(p > 0.05)$
**ET**		$(p > 0.05)$	-1.72 $(p = 0.0432)$	$(p > 0.05)$
**LSM**			-2.82 $(p = 2.38 \times 10^{-3})$	$(p > 0.05)$
**NS**				2.12 $(p = 0.0172)$

Academic 2021	**ET**	**LSM**	**NS**	**SSM**
**AH**	$(p > 0.05)$	-2.08 $(p = 0.0189)$	-1.65 $(p = 0.0497)$	$(p > 0.05)$
**ET**		-1.69 $(p = 0.0451)$	$(p > 0.05)$	$(p > 0.05)$
**LSM**			$(p > 0.05)$	1.79 $(p = 0.0448)$
**NS**				$(p > 0.05)$

H 2021	**ET**	**LSM**	**NS**	**SSM**
**AH**	$(p > 0.05)$	$(p > 0.05)$	$(p > 0.05)$	$(p > 0.05)$
**ET**		$(p > 0.05)$	$(p > 0.05)$	$(p > 0.05)$
**LSM**			-2.00 $(p = 0.0228)$	$(p > 0.05)$
**NS**				2.35 $(p = 9.34 \times 10^{-3})$

Academic 2022	**ET**	**LSM**	**NS**	**SSM**
**AH**	$(p > 0.05)$	-1.68 $(p = 0.0497)$	$(p > 0.05)$	$(p > 0.05)$
**ET**		$(p > 0.05)$	$(p > 0.05)$	$(p > 0.05)$
**LSM**			$(p > 0.05)$	$(p > 0.05)$
**NS**				$(p > 0.05)$

Citations 2022	**ET**	**LSM**	**NS**	**SSM**
**AH**	$(p > 0.05)$	1.69 $(p = 0.0454)$	$(p > 0.05)$	$(p > 0.05)$
**ET**		$(p > 0.05)$	-1.71 $(p = 0.0432)$	$(p > 0.05)$
**LSM**			-2.63 $(p = 4.25 \times 10^{-3})$	$(p > 0.05)$
**NS**				2.19 $(p = 0.0142)$

H 2022	**ET**	**LSM**	**NS**	**SSM**
**AH**	$(p > 0.05)$	$(p > 0.05)$	2.37 $(p = 8.85 \times 10^{-3})$	$(p > 0.05)$
**ET**		$(p > 0.05)$	-1.71 $(p = 0.0432)$	$(p > 0.05)$
**LSM**			-2.27 $(p = 0.0117)$	$(p > 0.05)$
**NS**				2.11 $(p = 0.172)$

Academic 2023	**ET**	**LSM**	**NS**	**SSM**
**AH**	$(p > 0.05)$	-1.65 $(p = 0.0497)$	-1.65 $(p = 0.0497)$	$(p > 0.05)$
**ET**		$(p > 0.05)$	$(p > 0.05)$	$(p > 0.05)$
**LSM**			$(p > 0.05)$	$(p > 0.05)$
**NS**				$(p > 0.05)$

Employer 2023	**ET**	**LSM**	**NS**	**SSM**
**AH**	-2.84 $(p = 2.16 \times 10^{-3})$	-1.65 $(p = 0.0497)$	-2.85 $(p = 2.16 \times 10^{-3})$	$(p > 0.05)$
**ET**		$(p > 0.05)$	$(p > 0.05)$	1.74 $(p = 0.0407)$
**LSM**			$(p > 0.05)$	$(p > 0.05)$
**NS**				1.79 $(p = 0.0368)$

Citations 2023	**ET**	**LSM**	**NS**	**SSM**
**AH**	$(p > 0.05)$	1.69 $(p = 0.0451)$	$(p > 0.05)$	1.74 $(p = 0.0411)$
**ET**		$(p > 0.05)$	-1.71 $(p = 0.0432)$	$(p > 0.05)$
**LSM**			-1.90 $(p = 0.0289)$	$(p > 0.05)$
**NS**				1.94 $(p = 0.0258)$

Table S3. Statistical z-tests on proportions to evaluate the significance of the differences between the fraction of subjects with a significant territorial bias ($10^{-s} < 0.05$), comparing two macro-areas for each combination of ranking type and year. Unreported combinations have no significant outcome. The alternative hypothesis is that the fraction of subjects reporting a statistically significant territorial bias is larger (smaller) for the macro-area on the rows, if the reported statistical parameter is positive (negative).

	Academic 2020	Academic 2021	Academic 2022	Academic 2023	Employer 2020	Employer 2021	Employer 2022	Employer 2023	Citations 2020	Citations 2021	Citations 2022	Citations 2023	H 2020	H 2021	H 2022	H 2023
**Arts and Humanities**	6	11	11	11	6	11	11	11	6	7	7	7	6	7	6	6
Archaeology	X	X	X	X	X	X	X	X	X	X	X	X	X	X	X	X
Architecture and Built Environment	X	X	X	X	X	X	X	X	X	X	X	X	X	X	X	X
Art and Design		X	X	X		X	X	X								
Classics and Ancient History		X	X	X		X	X	X								
English Language and Literature		X	X	X		X	X	X		X	X	X		X		
History	X	X	X	X	X	X	X	X	X	X	X	X	X	X	X	X
Linguistics	X	X	X	X	X	X	X	X	X	X	X	X	X	X	X	X
Modern Languages		X	X	X		X	X	X								
Performing Arts		X	X	X		X	X	X								
Philosophy	X	X	X	X	X	X	X	X	X	X	X	X	X	X	X	X
Theology, Divinity and Religious Studies	X	X	X	X	X	X	X	X	X	X	X	X	X	X	X	X
**Engineering and Technology**	7	7	7	7	7	7	7	7	7	7	7	7	7	7	7	7
Computer Science and Information Systems	X	X	X	X	X	X	X	X	X	X	X	X	X	X	X	X
Engineering - Chemical	X	X	X	X	X	X	X	X	X	X	X	X	X	X	X	X
Engineering - Civil and Structural	X	X	X	X	X	X	X	X	X	X	X	X	X	X	X	X
Engineering - Electrical and Electronic	X	X	X	X	X	X	X	X	X	X	X	X	X	X	X	X
Engineering - Mechanical	X	X	X	X	X	X	X	X	X	X	X	X	X	X	X	X
Engineering - Mineral and Mining	X	X	X	X	X	X	X	X	X	X	X	X	X	X	X	X
Engineering - Petroleum	X	X	X	X	X	X	X	X	X	X	X	X	X	X	X	X
**Life Sciences and Medicine**	8	9	9	9	8	9	9	9	8	9	9	9	8	9	9	9
Agriculture and Forestry	X	X	X	X	X	X	X	X	X	X	X	X	X	X	X	X
Anatomy and Physiology	X	X	X	X	X	X	X	X	X	X	X	X	X	X	X	X
Biological Sciences		X	X	X		X	X	X		X	X	X		X	X	X
Dentistry	X	X	X	X	X	X	X	X	X	X	X	X	X	X	X	X
Medicine	X	X	X	X	X	X	X	X	X	X	X	X	X	X	X	X
Nursing	X	X	X	X	X	X	X	X	X	X	X	X	X	X	X	X
Pharmacy and Pharmacology	X	X	X	X	X	X	X	X	X	X	X	X	X	X	X	X
Psychology	X	X	X	X	X	X	X	X	X	X	X	X	X	X	X	X
Veterinary Sciences	X	X	X	X	X	X	X	X	X	X	X	X	X	X	X	X
**Natural Sciences**	9	9	9	9	9	9	9	9	9	9	9	9	9	9	9	9
Chemistry	X	X	X	X	X	X	X	X	X	X	X	X	X	X	X	X
Earth and Marine Sciences	X	X	X	X	X	X	X	X	X	X	X	X	X	X	X	X
Environmental Sciences	X	X	X	X	X	X	X	X	X	X	X	X	X	X	X	X
Geography	X	X	X	X	X	X	X	X	X	X	X	X	X	X	X	X
Geology	X	X	X	X	X	X	X	X	X	X	X	X	X	X	X	X
Geophysics	X	X	X	X	X	X	X	X	X	X	X	X	X	X	X	X
Materials Sciences	X	X	X	X	X	X	X	X	X	X	X	X	X	X	X	X
Mathematics	X	X	X	X	X	X	X	X	X	X	X	X	X	X	X	X
Physics and Astronomy	X	X	X	X	X	X	X	X	X	X	X	X	X	X	X	X
**Social Sciences and Management**	13	13	15	15	13	13	15	15	13	13	15	15	13	13	13	13
Accounting and Finance	X	X	X	X	X	X	X	X	X	X	X	X	X	X	X	X
Anthropology	X	X	X	X	X	X	X	X	X	X	X	X	X	X	X	X
Business and Management Studies	X	X	X	X	X	X	X	X	X	X	X	X	X	X	X	X
Communication and Media Studies	X	X	X	X	X	X	X	X	X	X	X	X	X	X	X	X
Development Studies	X	X	X	X	X	X	X	X	X	X	X	X	X	X	X	X
Economics and Econometrics	X	X	X	X	X	X	X	X	X	X	X	X	X	X	X	X
Education and Training	X	X	X	X	X	X	X	X	X	X	X	X	X	X	X	X
Hospitality and Leisure Management			X	X			X	X			X	X				
Law and Legal Studies	X	X	X	X	X	X	X	X	X	X	X	X	X	X	X	X
Library and Information Management	X	X	X	X	X	X	X	X	X	X	X	X	X	X	X	X
Politics	X	X	X	X	X	X	X	X	X	X	X	X	X	X	X	X
Social Policy and Administration			X	X			X	X			X	X				
Sociology	X	X	X	X	X	X	X	X	X	X	X	X	X	X	X	X
Sports-Related Subjects	X	X	X	X	X	X	X	X	X	X	X	X	X	X	X	X
Statistics and Operational Research	X	X	X	X	X	X	X	X	X	X	X	X	X	X	X	X
Art History				X				X								
Data Science				X				X				X				X
Marketing				X				X				X				X

Table S4. Ranking availability for subjects, grouped by macro-area. The last three subjects are still officially unclassified. The rows with the macro-area names report the number of available rankings for each type and year.

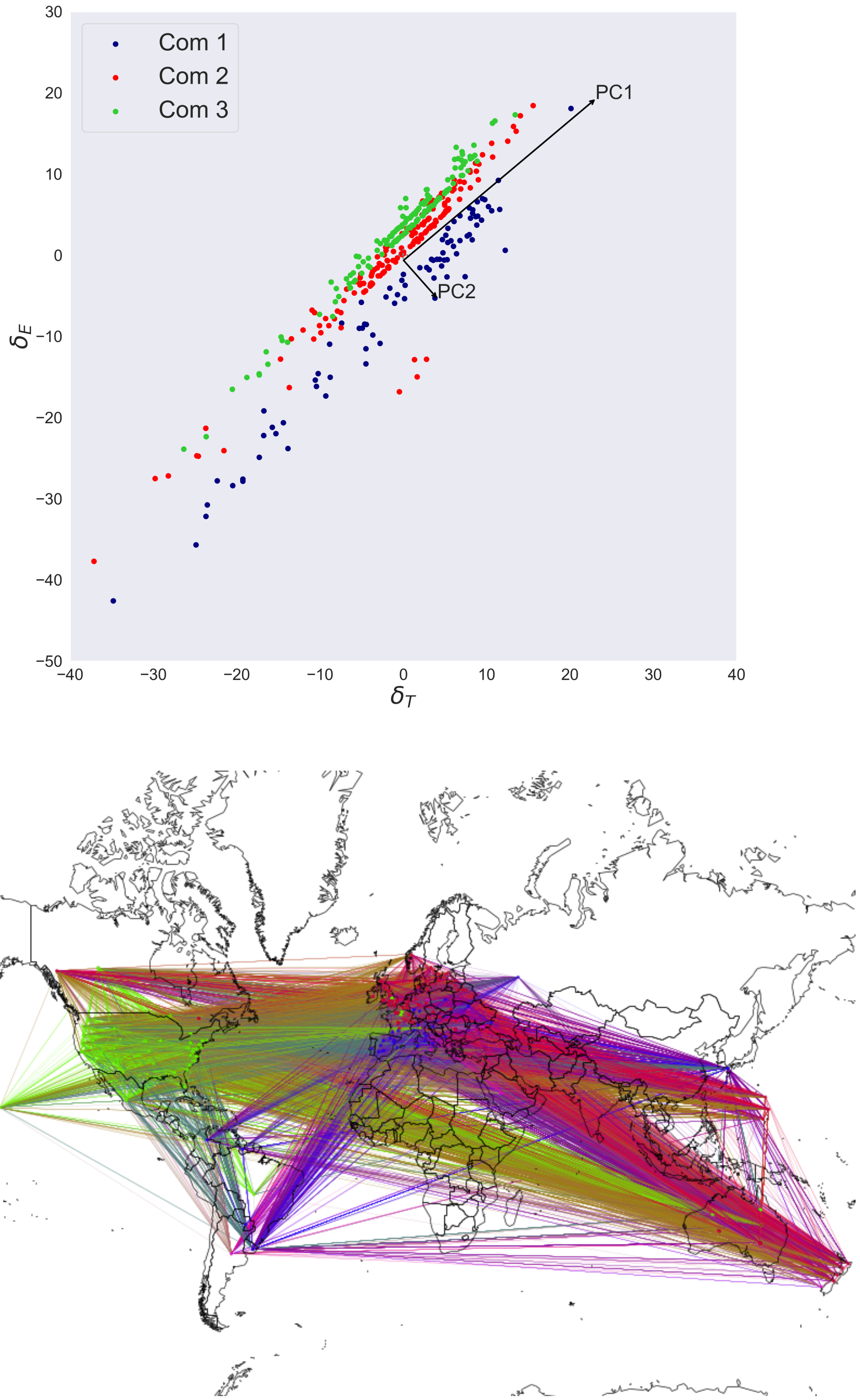

Fig S2. Debiasing procedure applied to the Citations ranking of the subject *Business and Management Studies* for the year 2020, characterized by the assortativity value 2.85×10^{-2}, with standard error 2.72×10^{-3} and significance score $s = 24.9$. The upper panel illustrates the distribution of the debiasing parameters (δ_T, δ_E) for all the universities listed in the ranking; each point represents a ranked institution and is coloured according to territorial community membership. The directions of the principal components PC1 (corresponding to the debiased score) and PC2 are reported in the plot. The lower panel shows a map of the links in the territorial network, on which the identification of territorial communities is based. The map is generated with the "Map of Countries" and "GeoLayout" plugins of Gephi 0.9.5 (Gephi Consortium, 2022, `https://gephi.org`).

**Ranking**	**Average** $\lvert\Delta r_w\rvert/S_w$	
	$p = 0.001$ vs $p = 0.01$	$p = 0.05$ vs $p = 0.01$
Academic 2020	0.31	0.27
Academic 2021	0.34	0.32
Academic 2022	0.26	0.22
Academic 2023	0.22	0.21
Citations 2020	0.67	0.72
Citations 2021	0.69	0.73
Citations 2022	0.69	0.75
Citations 2023	0.50	0.54
Employer 2020	0.30	0.23
Employer 2021	0.45	0.46
Employer 2022	0.41	0.33
Employer 2023	0.23	0.24
H 2020	0.53	0.59
H 2021	0.49	0.57
H 2022	0.47	0.59
H 2023	0.49	0.57

Table S5. Test of the statistical consistency between assortativities obtained with different acceptance thresholds of Pearson correlation statistical significance in the subregion networks. The table reports the values, averaged over the subjects, of the absolute discrepancy between assortativities r_w obtained with different thresholds, normalized to the standard error S_w for $p = 0.01$.

δ_E **Academic**	$\mathcal{G}_1$	$\mathcal{G}_C$	$\mathcal{G}_{0.75}$
$\mathcal{G}_C$	0.998		
$\mathcal{G}_{0.75}$	0.931	0.934	
$\mathcal{G}_{0.50}$	0.905	0.902	0.931
δ_E **Citations**	$\mathcal{G}_1$	$\mathcal{G}_C$	$\mathcal{G}_{0.75}$
$\mathcal{G}_C$	0.999		
$\mathcal{G}_{0.75}$	0.942	0.942	
$\mathcal{G}_{0.50}$	0.925	0.923	0.952
δ_E **Employer**	$\mathcal{G}_1$	$\mathcal{G}_C$	$\mathcal{G}_{0.75}$
$\mathcal{G}_C$	1.000		
$\mathcal{G}_{0.75}$	0.996	0.996	
$\mathcal{G}_{0.50}$	0.977	0.977	0.976
δ_E **H**	$\mathcal{G}_1$	$\mathcal{G}_C$	$\mathcal{G}_{0.75}$
$\mathcal{G}_C$	0.997		
$\mathcal{G}_{0.75}$	0.991	0.990	
$\mathcal{G}_{0.50}$	0.977	0.979	0.983

Table S6. Pearson correlations between pairs of δ_E rankings, related to each 2020 ranking of the subject *Business and Management Studies*, obtained from different configurations of the educational offer network. The graph $\mathcal{G}_1$ describes the network considered in the main text, constructed by starting from the maximum spanning tree associated to the complete graph of the educational offer network, and then progressively added other links, in decreasing order of weight, to minimize the gap between the densities of the educational offer network and the corresponding territorial network. The graph $\mathcal{G}_C$ describes the complete educational offer network. The graphs $\mathcal{G}_{0.75}$ and $\mathcal{G}_{0.50}$ are obtained with a similar procedure as $\mathcal{G}_1$, but requiring the network density to be the closest to the 75% and 50% of the territorial network density, respectively.
